# E-cigarettes and periodontal health: current evidence and research gaps

**DOI:** 10.1590/1807-3107bor-2026.vol40suppl1064

**Published:** 2026-08-21

**Authors:** Claudio Mendes PANNUTI, Thaís Emilia da SILVA, Sabrina Garcia de AQUINO, João Paulo STEFFENS, Gustavo Giacomelli NASCIMENTO, Fabio Renato Manzolli LEITE

**Affiliations:** (a) Universidade de São Paulo - USP, School of Dentistry, Department of Stomatology, São Paulo, SP, Brazil; (b) Universidade Federal da Paraíba - UFPB, Department of Clinical and Social Dentistry, João Pessoa, PB, Brazil; (c) Universidade Federal do Paraná - UFPR, Department of Stomatology, Curitiba, PR, Brazil; (d) University of Utah - School of Dentistry, Salt Lake City, UT, USA

**Keywords:** Periodontal Diseases, Smoking, Smoking Devices

## Abstract

Cigarette smoking is a well-established risk factor for periodontitis, contributing to increased clinical attachment loss (CAL), alveolar bone loss, and impaired treatment response. As electronic cigarettes (ECs) are increasingly used as alternatives to combustible tobacco, questions remain regarding their adverse effects on oral and periodontal health. This critical review integrated evidence from experimental, cross-sectional, longitudinal and interventional studies to evaluate the relationship between EC use and periodontal health. Experimental and molecular studies supported biological plausibility for periodontal harm, indicating that aerosols can induce oxidative stress, upregulate pro-inflammatory mediators, and disrupt host-microbial interactions. Cross-sectional studies generally reported poorer periodontal conditions among EC users compared with never-smokers, including higher plaque index and periodontal probing depth, although results for CAL and alveolar bone loss were inconsistent. Comparisons between EC users and cigarette smokers often showed more favorable clinical parameters among EC users. Longitudinal evidence continued to be limited. The only prospective cohort study reported higher CAL among EC users than never-smokers and observed an increase in CAL over time exclusively in EC users. Interventional studies assessing response to periodontal therapy have yielded inconsistent results, with some suggesting a poorer treatment response among EC users and others finding no significant differences. Overall, there were Well-designed longitudinal studies with clearly defined exposure groups, standardized periodontal outcomes, and longer follow-up are needed to clarify whether EC use independently contributes to periodontal deterioration.

## Introduction

Cigarette smoking is a major risk factor for periodontitis, is associated with its higher prevalence, extent, and severity,^1^,^2^,^3^,^4^ as well as with an increased rate of disease progression over time.^3^,^4^ Among heavy smokers, periodontal treatment has shown beneficial effects on severe periodontitis and was accompanied by ongoing periodontal destruction, evidenced by progressive clinical attachment loss (CAL throughout follow-up.^5^ Importantly, smoking cessation has been shown to result in periodontal probing depth(PPD) reduction and clinical attachment gain after periodontal therapy,^6^,^7^,^8^ supporting a causal relationship between smoking and periodontitis.

Although most smokers wish to quit, unassisted cessation attempts are associated with low long-term abstinence rates, as a result of psychological, behavioral, and physical dependence.^9^,^10^ Behavioral and pharmacological interventions, including nicotine replacement therapy and pain medications, have increased cessation success, but sustained abstinence rates have remained modest, typically around 30%.^10^

Within this context, electronic cigarettes (ECs) have emerged as an alternative nicotine delivery system, initially promoted as a harm-reduction strategy and as an aid for transitioning away from combustible tobacco products.^11^ ECs generate inhalable aerosols by heating e-liquids that may contain nicotine, flavoring agents, and other chemical compounds, thereby replicating several sensory and behavioral aspects of conventional smoking.^11^ Moreover, thermal degradation of e-liquids can generate toxic and carcinogenic by-products, including aldehydes, carbonyls, and metals.^12^ Consequently, concerns have been raised regarding the potential adverse effects of EC use, particularly on the respiratory and cardiovascular systems.^11^,^12^ Moreover, variability in product composition and limited regulatory oversight may result in nicotine exposure levels sufficient to induce and maintain nicotine dependence.^11^,^12^

The effects of ECs on oral health resemble those found for systemic health outcomes. Cross-sectional studies have shown poor periodontal health among EC users (also known as aim of vapers),^13^ although evidence from longitudinal studies continues to be limited. The aim of this review was to summarize and critically evaluate the current evidence on the association between EC use and periodontal health, highlighting methodological limitations, research gaps, and priorities for future investigation. Evidence examining the association between EC use and periodontal outcomes - derived from cross-sectional and longitudinal studies -was identified through electronic searches of PubMed via MEDLINE, Embase, Scopus, Web of Science, and the Cochrane Library databases. Details of the electronic search strategy are available in the Open Science Framework project files (https://osf.io/agr34/files/osfstorage). The electronic search was conducted up to February 2026, and only studies published in English were considered.

## Biological plausibility of EC effect on periodontal health

Experimental and clinical evidence indicated that shifts in host-microbial interactions and in microbial composition toward a dysbiotic profile partially overlapping with patterns described in conventional cigarette smokers, providing biological plausibility for potential periodontal effects of ECs. It continues to be uncertain whether these molecular and cellular alterations translate into clinically meaningful periodontal tissue damage.

Exposure to EC has been associated with changes in pro-inflammatory cytokine levels, although the magnitude and clinical relevance vary across studies. Elevated levels of interleukin (IL) -1β, IL-6, tumor necrosis factor-alpha (TNF-α), and interferon-gamma (IFN-γ) have been reported among vapers compared with those of non-smokers in saliva^14^,^15^ and in gingival crevicular fluid (GCF).^16.17^,^18^,^19^ Reductions in anti-inflammatory mediators, such as IL-10, and increased matrix metalloproteinase-8 (MMP-8) expression have also been observed in the GCF of vapers,^16^,^17^ suggesting a pro-inflammatory milieu and early collagen turnover.

Values of Cytokine profiles in the GCF and saliva of vapers were reported as being intermediate between those of cigarette smokers and non-smokers^16^,^17^,^18^,^19^,^20^ or similar to those of cigarette smokers.^15^,^19^ The majority of studies had a cross-sectional design, limiting causal inference relative to periodontal tissue destruction. Dual users showed greater inflammatory activation and altered bone metabolism markers than those of exclusive vape users, such as receptor activator of Nuclear Factor Kappa-B ligand (RANKL) and osteoprotegerin (OPG), indicating cumulative exposure effects and possible impacts on alveolar bone homeostasis.^17^,^21^

Saliva and subgingival plaque samples had enrichment of genera such as *Treponema* and *Filifactor,* with increased detection of *Fusobacterium nucleatum* and *Porphyromonas gingivalis* in cross-sectional studies.^14^,^15^,^22^ In a six-month prospective study, subgingival samples from vapers with periodontitis showed relative ecological stability, with persistent enrichment of *Porphyromonas, Treponema,* and *Veillonella* species, and high alpha diversity.^23^ Nevertheless, the clinical significance of these changes and the effects of dual use remain unclear.

In vitro studies indicated that EC aerosols increase oxidative stress and activate pro-inflammatory signaling in oral tissues, impairing local immune defenses.^24^,^25^,^26^,^27^ Proinflammatory cytokine upregulation and increased invasion by *P. gingivalis* and *F. nucleatum* in oral epithelial cells and periodontal fibroblasts were observed after exposure to EC aerosol^14^ Neutrophil dysfunction, including impaired sustained abstinence rates remained modest, phagocytosis and reduced antimicrobial activity, have been reported after exposure to EC aerosols, even in the absence of nicotine,^25^ as well as increased levels of cyclooxygenase-2 (COX-2), prostaglandin E2 (PGE2), and receptor for advanced glycation end products (RAGE) in gingival epithelial cells.^24^ These effects partially overlapped with oxidative stress mechanisms induced by conventional cigarette, depending on aerosol composition and chemical constituents.^25^,^26^,^27^ EC aerosols also disrupted gingival fibroblast homeostasis and impaired osteoblast proliferation and differentiation, associated with increased RANKL expression, reduced OPG secretion, and lower alkaline phosphatase activity.^26^,^28^

Evidence further indicated alterations in repair-related processes, including reduced proliferation and migration, increased apoptosis, DNA damage, and premature senescence in gingival epithelial and progenitor cells, induced by ECs aerosols or condensates.^19^,^24^,^26^,^27^ Both cigarette smoke and EC condensates seemed to impair gingival fibroblast function, although cytotoxicity was lower with ECs.^28^,^29^ Three-dimensional gingival mucosa models showed altered architecture and increased pro-inflammatory cytokine expression led by EC aerosols.^30^ Similarly, osteoblast exposure to e-liquids and vapor condensates was associated with disruption of signaling pathways involved in alveolar bone repair.^26^,^27^ Overall, these findings indicated that ECs disturb cellular pathways involved in periodontal and alveolar tissue repair. However, the absence of robust clinical and animal data limits conclusions relative to in vivo relevance. Figure summarizes the main biological mechanisms through which EC exposure may alter periodontal tissues, observed by *in vitro* studies. During the preparation of the schematic representation, the authors used a generative artificial intelligence text-to-image tool (ChatGPT, OpenAI, San Francisco, CA, USA) to assist in the creation ofvisual elements based on detailed author-developed prompts specifically designed to represent the biological and pathophysiological mechanisms discussed in the manuscript. All scientific content, conceptual organization, and final figure supervision were performed and verified by the authors.

**Figure:**
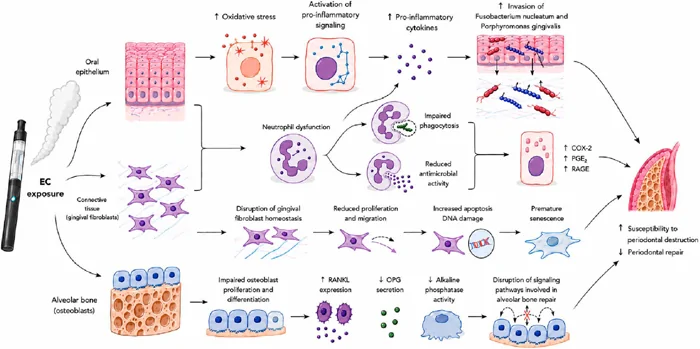
Schematic representation of the main biological mechanisms associated with electronic cigarette-induced periodontal tissue alterations observed in *in vitro* studies. Visual elements were generated with assistance from ChatGPT (OpenAI) using author-developed text prompts and subsequently reviewed and curated by the authors.

## Cross-sectional studies

### Vapers versus never-smokers

In cross-sectional studies, vapers presented poorer oral health and increased self-reported periodontal diseases, as well as higher gingival and community periodontal index scores, compared with never-smokers.^31^,^32^,^33^,^34^,^35^ Clinical periodontal parameters, including plaque index (PI)^21^,^36^,^37^ and mean PPD^21^,^36^,^37^,^38^ were generally higher in vapers, despite the absence of statistically significant differences.^38^,^39^

Findings for CAL and alveolar bone loss (ABL) were inconsistent. Some studies reported higher CAL and ABL among vapers,^16^,^32^,^36^,^38^ whereas others found no differences compared with never-smokers.^37^,^39^ Such heterogeneity probably reflects variations in study populations, exposure definitions, device characteristics, aerosol composition, outcome assessments, and residual confounding factors.

Lower percentages of bleeding on probing (BOP) have been reported among vapers,^16^,^37^ resembling findings from cigarette smokers. This pattern may reflect smoking-related modulation of the inflammatory and vascular response rather than improved periodontal health, potentially masking underlying tissue inflammation. Overall, while cross-sectional studies suggested associations between EC use and selected periodontal parameters, the cross-sectional nature of the evidence precludes causal inference and hinders conclusions regarding the direction and clinical relevance of these findings.

### Vapers versus conventional cigarette smokers

Cross-sectional comparisons between vapers and cigarette smokers have generally reported more favorable clinical parameters among the former. Specifically, lower gingival and PI scores and fewer sites with PPD ≥ 6 mm have been observed in vapers compared with conventional smokers.^40^,^41^ However, findings for CAL were inconsistent, with some studies reporting lower values among vapers,^40^ whereas others found no differences.^36^,^37^ Importantly, interpretation of these comparisons is constrained by differences in exposure history between groups. Cigarette smokers typically have longer and more intense cumulative exposure to tobacco products than vapers, many of whom are former smokers or dual users. The differences observed may reflect disparities in cumulative exposure rather than intrinsic differences in the biological effects of ECs versus conventional cigarettes.

### Longitudinal studies

Four prospective interventional studies,^42^,^43^,^44^,^45^ one retrospective interventional study,^46^ and one prospective cohort study^47^ were identified. A table summarizing the longitudinal studies and the full electronic search strategy are available in the Open Science Framework repository (https://osf.io/agr34/files/osfstorage). The majority of longitudinal studies evaluated periodontal treatment response rather than the natural history or incidence of periodontitis. Therefore, their findings primarily informed how tissues responded to therapy under different exposure conditions, rather than establishing causal effects of EC use on periodontitis development.

### Interventional studies

In all interventional studies, participants received the same non-surgical periodontal therapy within a single treatment arm. Comparisons were based on exposure status (EC users, cigarette smokers, and never-smokers), and inter-group differences at baseline and follow-up, as well as intra-group changes over time.

At baseline, no differences were observed between vapers and never-smokers regarding CAL, PI,^42-45^ and ABL.^42^,^44^ In one study, baseline PPD was significantly higher in vapers than in never-smokers,^45^ while in others, BOP was higher in never-smokers than in vapers.^42^,^43^,^44^ At follow-up, vapers presented significantly greater mean PPD and PI scores than never-smokers.^42^,^44^ Some studies observed greater reductions in BOP and mean PPD among never-smokers after treatment,^42^,^43^ while others found no significant differences in treatment response between the groups over time.^44^,^45^

Two studies compared vapers and cigarette smokers,^43^,^44^ and found no differences between groups for any periodontal parameter at baseline. At follow-up, one study detected higher mean PPD and PI in smokers than in vapers,^43^ whereas the other reported no significant inter-group differences.^44^ Relative to intra-group changes, one study identified smaller reductions among cigarette smokers,^43^ while the other found no differences in treatment response between groups.^44^

In the retrospective interventional study,^46^ baseline periodontal parameters did not differ between vapers and never-smokers. At follow-up, CAL and PPD were significantly higher in vapers, and analyses suggested a less favorable response to non-surgical periodontal therapy among vapers. Overall treatment response patterns were similar between groups. Interpretation of these comparisons is constrained by the retrospective design and differences in cumulative history of exposure to tobacco products.

### Cohort study

The only prospective cohort study identified^47^ compared periodontal outcomes among vapers, cigarette smokers, and never-smokers. At baseline, cigarette smokers presented with a higher prevalence of severe periodontitis, although this difference was largely confounded by age. CAL was higher in vapers than among never-smokers and higher in cigarette smokers than among vapers. During follow-up, mean BOP and PPD increased over time in all groups. A significant intra-group increase in CAL over time was observed exclusively among vapers. However, this observation was derived from a relatively short follow-up period and exposure group definitions that may not have accounted fully for prior smoking history. Accordingly, this finding should be interpreted cautiously.

## Discussion, research gaps, and future perspectives

This review integrated evidence from multiple study designs to evaluate the relationship between EC use and periodontal health. Experimental and molecular studies showed that EC aerosols were associated with deleterious effects on oral cells, including oxidative stress, changes in proinflammatory mediators, and host-microbial interactions.^14^,^17^,^23^,^48^ These findings supported biological plausibility for periodontal effects of vaping and indicated that EC aerosols were not inert at the cellular level. However, their relevance probably depends on the magnitude, frequency, and duration of exposure, plus on the extent to which aerosol constituents reached target sites within tissues.

While cellular alterations appeared to exist, their translation into clinically relevant periodontal outcomes remained uncertain. Evidence was limited and heterogeneous, with the majority of data derived from cross-sectional studies. Although studies reported associations between EC use and some clinical parameters, results were inconsistent, particularly for indicators of cumulative tissue breakdown such as CAL^.16^,^36^,^37^ Given the multicausal etiology of periodontitis and the relatively recent and heterogeneous nature of EC exposure, possible modest or context-dependent effects may have been difficult to detect.

Interpretation of periodontal parameters is complicated by smoking-related modulation of inflammatory and vascular responses. Low levels of BOP among vapers have been reported, a pattern also observed in cigarette smokers.^16^,^37^ Reduced BOP may reflect a modified clinical presentation of gingival inflammation rather than the absence of underlying inflammatory activity, thereby reducing the sensitivity of bleeding-based measures for assessing inflammation. This limitation may partially explain discrepancies between clinical and molecular evidence of altered inflammatory mediator levels and bone metabolism markers in saliva and GCF.^18^,^21^

Methodological limitations hindered conclusions. Exposure assessment was frequently based on self-reported vaping status, with limited characterization of exposure duration, intensity, device type, or liquid composition. Many individuals classified as EC users were former smokers or dual users, complicating attempts to isolate the independent effects of vaping from cumulative exposure to combustible tobacco products.^(17)^ Comparisons between vapers and cigarette smokers were particularly challenging, as these groups were rarely exchangeable with respect to their sociodemographic characteristics as well as lifetime exposure, limiting causal interpretation of findings suggesting more favorable periodontal parameters among vapers.^40^,^41^ Future studies would benefit from more detailed exposure characterization, including duration and frequency of EC use, differentiation between exclusive and dual users, detailed reporting of device characteristics and liquid composition, and, when feasible, biochemical assessment of nicotine exposure, since labeled nicotine concentrations may not accurately reflect actual exposure. Longitudinal studies are scarce and the majority evaluate response to non-surgical periodontal therapy rather than disease incidence or progression, primarily informing short-term tissue responses under controlled clinical conditions and undermining the extrapolation of results.^42^,^43^,^44^ This design does not permit conclusions regarding the etiologic effects of EC use on periodontitis development. Only one prospective cohort study examined periodontal changes over time among vapers, reporting an increase in CAL during follow-up.^47^ Interpretation of this finding was constrained by limited follow-up for a disease with a slow progression rate in the majority of cases, baseline imbalances between exposure groups, and the aforementioned limited granularity in exposure characterization. Longer follow-up periods will be important to detect clinically meaningful effects in future studies, considering that periodontitis is a chronic disease with slow progression and EC exposure is relatively recent. The persistence of these gaps in evidence probably reflected broader structural challenges in periodontal and smoking-related research. Well-designed longitudinal studies require substantial financial resources, extended follow-up, and stable exposure definitions, particularly in the context of rapidly evolving products and heterogeneous regulation worldwide. In addition, identifying and retaining sufficiently large samples of exclusive vapers with minimal prior smoking exposure remains logistically challenging. These constraints may partially explain the predominance of cross-sectional analyses and short-term interventional studies.

Within the context of these methodological limitations and persistent gaps in evidence, it is also important to clarify that although ECs were initially promoted as alternatives to combustible tobacco products, the current body of evidence does not support electronic cigarette use as a smoking cessation strategy, particularly given the scientific uncertainties regarding their long-term effects on periodontal and systemic health. Accordingly, smoking cessation counseling should be based on behavioral and pharmacological interventions rather than substitution with alternative nicotine delivery systems.

## Conclusions

Taken together, the current literature does not provide sufficient evidence to establish a causal relationship between EC use and periodontal tissue destruction. The absence of robust evidence of harm should not be interpreted as evidence of periodontal safety. Rather, the available data indicate scientific uncertainty characterized by biological plausibility, suggestive but inconsistent clinical associations, and substantial methodological limitations.

Advancing understanding in this field will require cohort studies with adequate follow-up to assess the incidence and progression of periodontal diseases among clearly defined exposure groups. Priority should be given to differentiating never-smokers, former smokers, exclusive vapers, and dual users, alongside standardized and objective exposure assessment. Harmonization of periodontal outcome definitions and integration of clinical, radiographic, and biological end points will be essential to strengthen inference and move toward accurate, evidence-based conclusions relative to effects of EC use on periodontal health.

## Data Availability

The authors declare that all data generated or analyzed during this study are included in this published article.
